# Plasmocytome costal solitaire: à propos d'un cas

**DOI:** 10.11604/pamj.2014.18.179.2550

**Published:** 2014-06-24

**Authors:** Narindra Njarasoa Mihaja Razafimanjato, Manjakaniaina Ravoatrarilandy, Andriamihaja Jean Claude Rakotoarisoa, Rodrigue Hasiniatsy, Allen Francis Hunald, Auberlin Felantsoa Rakototiana, Florine Rafaramino, Hanitrala Jean Louis Rakotovao

**Affiliations:** 1 Service de Chirurgie Thoracique, CHU/JRA, Antananarivo, Madagascar; 2 Service d'Oncologie, CHU/JRA, Antananarivo, Madagascar; 3 Service de Chirurgie Pédiatrique, CHU/JRA, Antananarivo Madagascar; 4 Service d'Urologie, CHU/JRA, Antananarivo, Madagascar

**Keywords:** Cote, paroi thoracique, plasmocytome osseux solitaire, plasmocytome costal, tumeur osseuse, rib, chest wall, solitary bone plasmacytoma, rib plasmacytoma, bone tumor

## Abstract

Les auteurs rapportent un cas de plasmocytome solitaire particulière par leur localisation costale. Le diagnostic est basé sur la mise en évidence d'une tumeur localisée, constituée de cellules plasmocytaires monoclonales cytologiquement identiques à celles du myélome multiple, en l'absence d'autres signes en faveur d'une forme disséminée. Nous rapportons un cas de plasmocytome solitaire à localisation costale et nous discutons les aspects diagnostiques et thérapeutiques de cette affection potentiellement menacée dans son évolution par la transformation en myélome multiple.

## Introduction

Le plasmocytome solitaire osseux (POS) est une entité rare, résultant de la prolifération de plasmocytes malins dérivant d'un clone unique de lymphocytes B, localisée à un endroit circonscrit de l'organisme sans envahissement médullaire diffus [1–4]. Il intéresse surtout le rachis dorsolombaire. L'atteinte costale est rarement décrite [1, 2]. Les auteurs rapportent un cas clinique et discutent les particularités diagnostiques et évolutives de cette pathologie.

## Patient et observation

**Figure 1:**
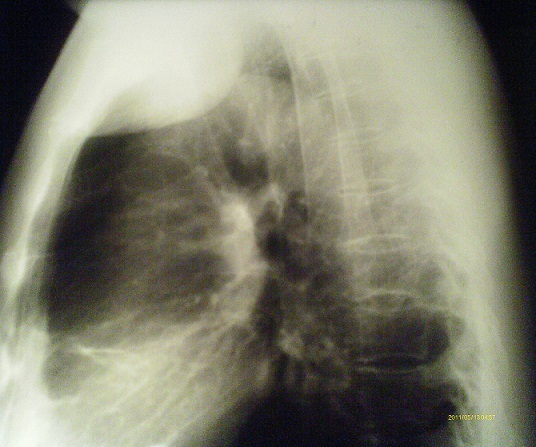
Radiographie du thorax en incidence de profil: Masse unique avec lyse costale massive et extension extra-pleurale et dans les parties molles

**Figure 2:**
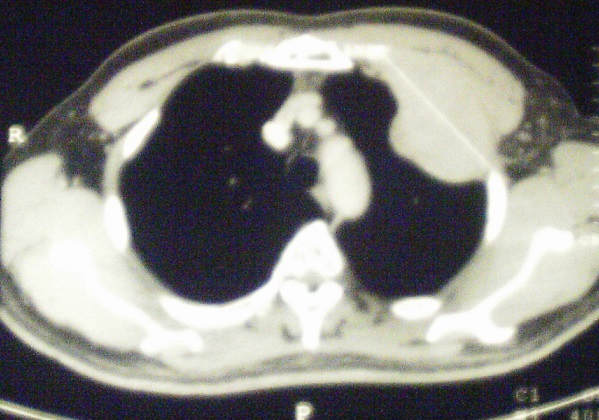
Scanner thoracique avec injection de produit de contraste en coupe transversale montrant une tumeur pariétale correspondant au plasmocytome costal solitaire

**Figure 3:**
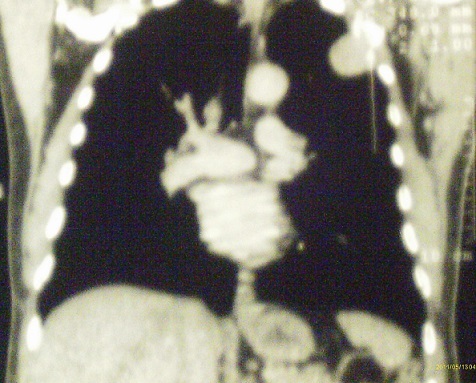
Scanner thoracique en coupe coronale masse développée au dépend de la paroi thoracique

**Figure 4:**
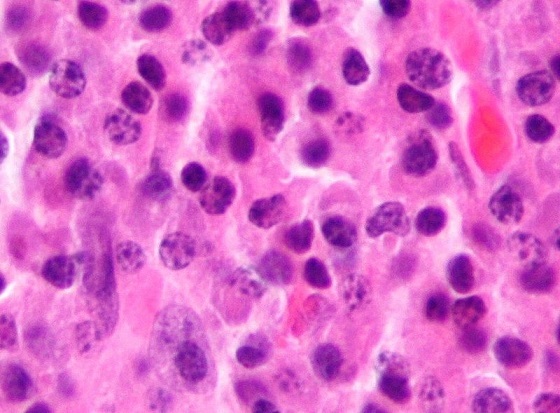
M/E, HE x 100: les cellules tumorales ont un aspect monomorphe, ce sont de grandes cellules à cytoplasme basophile, à noyau arrondi avec un nucléole volumineux en position centrale. Les cellules ressemblent à des plasmoblastes. De rares plasmocytes matures ont été observés au sein de la population cellulaire

RB, Malgache, âgé de 42 ans, non tabagique, sans antécédents particuliers a été hospitalisé au service de chirurgie thoracique pour tuméfaction pariétale douloureuse localisée au niveau de la région sous claviculaire gauche évoluant depuis six mois avant son admission. L'examen physique retrouvait un comblement du creux sus-claviculaire gauche sans adénopathies périphériques palpables. La radiographie thoracique révélait la présence d'une opacité tissulaire ayant un contact pariétal de 8 cm de grand axe (Figure 1). La tomodensitométrie thoracique objectivait une masse tumorale siégeant au niveau 2ème arc costal antérieur gauche se rehaussant après injection du produit de contraste avec déminéralisation osseuse (Figure 2, Figure 3). Le patient bénéficiait d'une segmentectomie du 2ème arc costal antérieur emportant la tumeur de 8 cm x 5 cm x 2 cm. L'examen histologique de la pièce opératoire a conclu à un plasmocytome malin (Figure 4). La biopsie ostéomédullaire ne montrait pas d'infiltration myélomateuse. Le bilan radiologique osseux, le dosage pondéral des immunoglobulines sériques, la numération formule sanguine, la fonction rénale et la calcémie étaient normaux. Le diagnostic de plasmocytome solitaire costal était retenu. La suite opératoire était simple. Six cures de chimiothérapie à base de Cyclophosphamide, d'Adriamycine, de Prednisone et de Vincristine étaient instituées (Protocole CAPV) Le patient est actuellement en rémission complète après un recul de trois ans.

## Discussion

Le plasmocytome solitaire osseux est défini par l'existence d'un infiltrat localisé de plasmocytes malins; sans atteinte plasmocytaire proliférative systémique ou disséminée [5]. Il se distingue par son caractère isolé, local et son évolution souvent lente [6]. C'est une tumeur rare, représentant moins de 5% des néoplasies à plasmocytes (PPM: Prolifération Plasmocytaire Maligne) [2, 5]. Il est exceptionnel dans sa localisation costale [5, 7]. C'est la troisième tumeur osseuse maligne de la paroi thoracique avec 20% de localisations dans cette région [8]. L’étiologie des plasmocytomes solitaires osseux reste inconnue. Des facteurs génétiques, l'exposition aux radiations, la stimulation antigénique chronique sont avancés dans la littérature comme facteurs de risques mais aucune association n'a jamais été prouvée [9]. Pour notre cas, nous n'avons retrouvé ni une exposition particulière, ni un antécédent marquant.

L’âge de notre patient se situe dans la tranche d’âge la plus fréquemment rapportée dans la littérature, à savoir entre 40 et 50 ans, dix ans de moins approximativement que pour le myélome multiple [6]. La prédominance masculine est nette, avec un sex-ratio de 3 à 4. [2, 5, 8].

Le plasmocytome solitaire osseux peut se manifester par des douleurs pariétales parfois liées à des fractures costales pathologiques ou une tuméfaction osseuse, comme pour notre patient, ou être de découverte radiologique fortuite [5]. Les formes asymptomatiques sont plus rares mais possibles [3]. En effet, ni les symptômes ni l'aspect macroscopique de la tumeur n'orientent pas d'emblée vers le plasmocytome; bien au contraire, ils contribuent souvent à un retard diagnostique non négligeable [10].

Sur le plan biologique, le plasmocytome solitaire se différencie du myélome multiple par un hémogramme normal, un myélogramme normal et une immunoélectrophorèse des protides mettant en évidence une sécrétion de protéines anormales, mais de faible taux [6]. Son aspect radiographique est celui d'une métastase avec une lacune ostéolytique costale à l'emporte-pièce ou fracture pathologique [8]. Certaines formes condensantes peuvent se rencontrer dans cadre de syndrome de POEMS (poly neuropathie, organomégalie, endocrinopathie, protéine monoclonale, anomalies cutanées) [3]. Au scanner, la masse présente un épaississement du cortex osseux avec insufflation, sclérose et effraction du cortex osseux [5]. L'imagerie par résonance magnétique (IRM) nucléaire est l'examen d'imagerie de référence. A part la lyse osseuse, il permet le diagnostic des atteintes médullaires diffuses et focales du MM (Myélome multiple) ainsi que pour les lésions extra-osseuses [5, 11]. Elle est particulièrement intéressante pour rechercher des anomalies précoces du signal médullaire, notamment lorsque le bilan radiologique standard ne révèle pas de lésions ostéolytiques [11]. Son intérêt en région costale n'a cependant pas été rapporté [3]. La Tomographie par Emission de Positons (TEP-TDM) au 18 FDG est capable de localiser des zones plus actives que d'autres, permettant ainsi au chirurgien d'adapter l’étendue de la résection et au oncologue de détecter précocement une éventuelle récidive ou transformation myélomateuse [8, 11]. Au total, le bilan d'imagerie, véritable « cartographie tumorale », précise la topographie de la tumeur, son extension, sa vascularisation et, plus largement, la resécabilité. Il permet d’évaluer l’étendue nécessaire de l'exérèse, les dangers préopératoires, et de prévoir les techniques de réparation pariétale. La confirmation histopathologique du plasmocytome solitaire osseux peut se faire par biopsie, par excision partielle ou par résection tumorale totale [5]. Dans notre cas, le diagnostic a été avancé par le résultat anatomopathologique inattendu de la pièce opératoire. Le diagnostic de plasmocytome solitaire osseux repose ainsi sur la preuve histologique de prolifération plasmocytaire, l'absence de diffusion médullaire; le caractère unique et localisé de la lésion ou lésion multiple sans avoir les critères de myélome multiple [11, 12]. Le plasmocytome est une tumeur radiosensible. Le traitement est basé sur une exérèse chirurgicale complète associée à une radiothérapie locale postopératoire [6, 8]. La dose administrée varie de 35 à 50 Gy en 15 à 25 séances sur 3 à 5 semaines. Ceci permet d'obtenir un contrôle local entre 86 à 100% des cas [5, 11].

Faute de radiothérapie, notre patient avait bénéficié d'une chimiothérapie adjuvante. Dans la littérature, la place de la chimiothérapie reste à définir [12]. Leur rôle dans la prévention de l’évolution vers la forme généralisée (MM) demeure un sujet de controverses [5, 8]. Une résection tumorale complète en premier, à viser à la fois diagnostique et thérapeutique complétée par une chimiothérapie, nous a permis d'obtenir une rémission complète avec un recul actuel de trois ans. L’évolution peut se faire vers la récidive locale ou le passage à la forme généralisée, dans 5 à 58% des cas avec un délai moyen de 2 à 3 ans [5, 8]. Elle s'accompagne fréquemment de nouvelles lésions osseuses et de nouveaux plasmocytomes osseux [11]. Avec la progression de la maladie, la protéine monoclonale peut être présente dans le sérum et les urines dans 24 à 72% des cas [5]. Un dosage de la protéinurie de Bence-Jones négatif nous a permis d’éliminer cette transformation myélomateuse pour notre cas.

## Conclusion

Le plasmocytome solitaire osseux dont le pronostic est favorable dans la majorité des cas, est une forme rare de néoplasie à plasmocytes, obéissant à certains critères diagnostiques. Nous approuvons encore à Madagascar la chirurgie pour l'obtention du diagnostic histologique, assurant par là le traitement car en absence de la radiothérapie une chirurgie radicale couplée à une chimiothérapie peut être une alternative thérapeutique. Le suivi des patients doit être prolongé afin de déceler précocement toute récurrence ou dissémination.
